# The Whistling Nuisance

**Published:** 1916-01-15

**Authors:** 


### THE WHISTLING NUISANCE.

It seems to be an undoubted legal right for any London householder to throw open his front door at any hour of the day or night and either personally, or through the agency of a servant, to utter frequent and strident blasts on a whistle for the purpose of attracting the attention of the driver of a taxi-cab. Moreover, the entertainment may be continued for an indefinite period, and as a matter of fact is continued until either the vehicle arrives or the performer is exhausted. Such is the patience of our citizens that up to the time of writing this practice has in no single instance produced any record of either battle or murder or sudden death. But times have changed, and while we hope that this fair record will not be stained by the bludgeon or bullet of some exasperated householder, the shrill whistler had better take heed to his steps. There are unmistakable signs that the limits of human endurance have been reached. Letters in large type have appeared in the *Times* and, still more, a question has been asked in the House of Commons.

These are certain evidences of a gathering storm, and it is to be feared that unless the shrieking is abated either ears will be nailed to the pump or we shall come under the terrors of penal legislation. While the double whistle for a hansom was formerly supported with equanimity, the single blast for the taxi-cab is no longer to be borne. The darker streets have encouraged us to earlier hours, and possibly our nerves are more tense under the stress of war. Cab-drivers, again, are fewer in number, and perhaps, like the rest of us, have learned the pleasures of the domestic hearth. Still more, our hospitals and nursing homes are full of wounded soldiers whose sufferings have a universal appeal, and to whom the insistent whistle often forbids the much needed quiet and sleep.

Such, broadly stated, are the circumstances which have determined not a few minds to the conclusion that a nuisance exists and that it ought to be abated. Voluntary measures are recommended, but if these fail, compulsion, here as in other directions, is to be invoked; and in this latter event there are to be no half-measures. It is true that advice has been given to the sufferers and to the sleepless to accommodate themselves to the necessary habits of a great and glorious city. One learned gentleman boldly advises retiral to bed only at an advanced hour of the night, while others urge the virtues of ear-pads, or of cotton-wool, or of wax. But it is vain to expect that these ingenious expedients will meet the position. It has come to be a question between the right of one individual to whistle and the right of another individual to sleep, and if the quarrel is forced into the legislative arena there can be little doubt of the choice of our senators. Hence both in the literal and figurative sense our whistlers had better go softly or they may find that their ear-splitting instrument has been rendered mute and that one more of our British liberties has been snatched from our hands.

